# Reasons for Taking the COVID-19 Vaccine by US Social Media Users

**DOI:** 10.3390/vaccines9040315

**Published:** 2021-03-29

**Authors:** Arriel Benis, Abraham Seidmann, Shai Ashkenazi

**Affiliations:** 1 Faculty of Industrial Engineering and Technology Management, Holon Institute of Technology, Holon 5810201, Israel; 2 Faculty of Digital Technologies in Medicine, Holon Institute of Technology, Holon 5810201, Israel; 3 Department of Information Systems, Questrom Business School, Boston University, Boston, MA 02215, USA; avis@bu.edu; 4 Health Analytics and Digital Health, Digital Business Institute, Boston University, Boston, MA 02215, USA; 5 School of Medicine, Ariel University, Ariel 40700, Israel; shaias@ariel.ac.il

**Keywords:** coronavirus, Sars-Cov-2, social media, online social networking, social factors, immunization programs, vaccination, vaccination refusal, vaccination coverage, vaccination hesitancy, health communication, health policy

## Abstract

Political and public health leaders promoting COVID-19 vaccination should identify the most relevant criteria driving the vaccination decision. Social media is increasingly used as a source of vaccination data and as a powerful communication tool to increase vaccination. In December 2020, we performed a cross-sectional social media-based survey addressing personal sentiments toward COVID-19 vaccination in the USA. Our primary research objective is to identify socio-demographic characteristics and the reasons for the 1644 survey participants’ attitudes regarding vaccination. We present clear evidence that, contrary to the prevailing public perceptions, young audiences using social media have mostly a positive attitude towards COVID-19 vaccination (81.5%). These younger individuals want to protect their families and their relatives (96.7%); they see vaccination as an act of civic responsibility (91.9%) and express strong confidence in their healthcare providers (87.7%). Another critical factor is the younger population’s fear of personal COVID-19 infection (88.2%); moreover, the greater the number of children the participants have, the greater is their intent to get the COVID-19 vaccine. These results enable a practical public-messaging pathway to reinforce vaccination campaigns addressing the younger population.

## 1. Introduction

### 1.1. Background

Since the introduction of the first modern vaccine, this inoculation process has faced public fears and opposition [1,2]. Consequently, each year around 50,000 people in the USA [3] and 1.5 million worldwide die due to vaccination hesitancy [4]. A wide range of variables may interact and lead to this situation [5]. One source of low adherence to vaccination is poor health literacy, which is significantly influenced by social media use [6].

**Figure 1:**
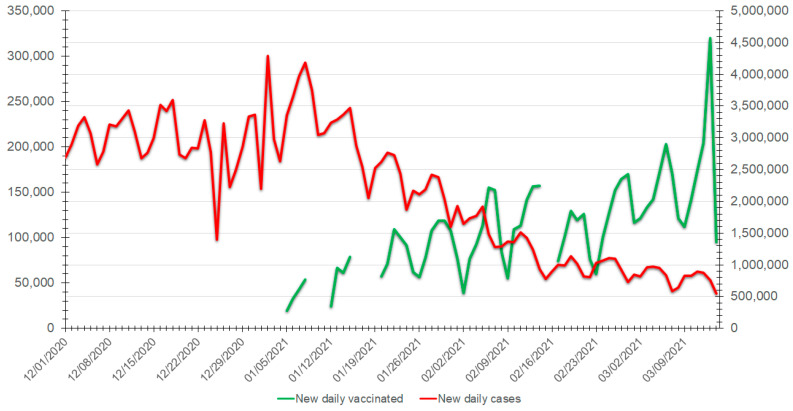
Evolution over time (between 1 December 2020 and 15 March 2021) of the number of new daily positive COVID-19 cases (left axis, red line) versus the number of new daily individuals vaccinated against COVID-19 (right axis, green line).

Dealing with vaccination hesitancy is a considerable challenge for healthcare and political decision-makers worldwide. Since January 2020, more than two million people have perished by COVID-19, with more than 100 million infected, many suffering from various severe symptoms and sequelae [7]. The rapid development and emergency approval of several COVID-19 vaccines by national and international agencies [8] have increased skepticism and hesitancy [9]. Although vaccines against COVID-19 are currently available in many countries, the overall vaccination adherence and vaccine coverage rates are relatively low, despite healthcare professional organizations’ recommendations, as has been witnessed with other vaccine-preventable diseases. The abundant misinformation about COVID-19 and the vaccines’ potential risks plays a considerable role in vaccine hesitancy. Therefore, public health policymakers need to understand which factors influence vaccination adherence or hesitancy for each population segment and adapt their communication accordingly. The positive impact of vaccination against COVID-19 in the USA is demonstrated (Figure 1) [10].

As with other vaccine-preventable diseases, communication and education have become notable vectors for reducing the disease’s effects. These are key prevention, response, and mitigation factors. When dealing with public health issues, it is crucial to consider epidemiological data, infodemiology, and infoveillance as a whole [11,12]. Immediate leadership actions are required, but enhancing the public’s COVID-19 literacy is challenging [9].

The communication and education landscapes are intimately related to the Internet, which is the primary vehicle for sharing information and knowledge in most societal domains, particularly among younger groups [13]. Indeed, the Internet is a new social determinant of health [14]. More specifically, online social networks and online social media are well-established communication tools.

The COVID-19 pandemic and the newly available vaccines against it emphasize the need to understand the reasons for vaccination adherence or hesitancy. Surveys over social media enable prompt recognition of the variables that may affect populations’ immunization decisions [15]. On the other hand, the traditional approaches for assessing public opinions rely on personal, telephone, or mail surveys. These can take a long time and thus are far less helpful for timely investigation during a rapidly evolving medical crisis. A large number of health-related surveys have been run over the Internet in recent years. Of course, this introduces a “social” bias in the investigated population. Nevertheless, such an approach can help policymakers to plan wisely the targets of health communication campaigns and reduce the risk and impacts of “infodemic” spread [16]. Understanding the behavioral underpinnings of an intent to adhere to COVID-19 vaccination among those who are not explicitly targeted participants can give insight into improving online communication about vaccination and increasing adherence.

### 1.2. Aim, Objectives, and Hypotheses

Our primary aim in this research was to elucidate social media users’ reasons for adherence to COVID-19 vaccination so as to devise social media interventions to increase vaccination rates, with potential application to other outbreaks [17]. The research’s primary objectives were to identify the reasons for survey participants’ disparate attitudes toward vaccination and to identify socio-demographic and other key attributes affecting COVID-19 vaccination adherence.

This cross-sectional survey-based research is led by two hypotheses that suggest that COVID-19 vaccination adherence is affected by:

1. A limited number of well-defined social variables;
2. Specific personal reasons.

## 2. Materials and Methods

The ongoing COVID-19 pandemic and public hesitancy regarding its related vaccines have led to increasing public health concerns. We focused on trying to understand the reasons motivating vaccination adherence or vaccination hesitancy.

### 2.1. Study Design and Participants

During a period of 15 days, from 10 December to 24 December 2020, we performed, over social media, a cross-sectional survey about COVID-19 vaccination intentions (Supplementary Material). The survey period coincided with the third COVID-19 pandemic wave and the approvals of the COVID-19 vaccines in the United States and other countries. The survey was built and run over Microsoft Forms and hosted by the principal investigator’s institution. The survey address was shared by posting it on various social media platforms (Facebook (Facebook Inc., Menlo Park, CA, USA), Reddit (Reedit Inc., San Francisco, CA, USA), Twitter (Twittter Inc., San Francisco, CA, USA), LinkedIn (Microsoft Copr., Sunnyvale, CA, USA), Google Ads (Google LLC, Mountain View, CA, USA)) and specifically in groups discussing at least one of the following subjects: COVID-19, vaccines, health, wellness, business, education, news at the local or national levels. 

Moreover, the survey link was sent via email to personal and professional contact lists. Some participants shared the survey address broadly as well.

The different social media channels used for spreading the Survey questionnaire are practical communication tools. However, each one has its specificity and population target.

- Facebook is a large public-focused, polyvalent platform, mainly used by 25–34-year-olds. Around two-thirds of its users in the USA log in daily, and more than a third declares that they get their news from Facebook. The average engagement rate per Facebook post is 0.27% [18,19].
- Reddit is a social media platform providing news aggregation, web content ratings, and blogging services. Moreover, the different communities are organized around specific subjects or foci (e.g., local or national news). In contrast with the other social media platforms, which are most often used via mobile applications, more than 50% of the Reddit users access it via a desktop computer. This means that they are more reactive to calls for action than mobile users, who are reviewing their message threads and are less available to answer a complex request such as a multi-question survey [20].
- Twitter is a microblogging platform. The largest age contingent among its users comprises 35–49-year-olds. The average engagement rate per Twitter post is 0.07% [18].
- Instagram is a photo and video sharing network used by less than half of adults in the USA. It is most popular among 25–34-year-olds.
- LinkedIn is a professional networking platform, so the most significant part of the activities on the site focus on individual and business promotion. The population accordingly is older than on Facebook. Around 25% of the US population uses this platform, and 6% use it as a news provider. LinkedIn is also mainly used by 25–34-year-olds.
- Email is not social media, per definition. Nevertheless, it allows sharing media content in both targeted and untargeted ways. Engagement depends on many personal and content factors.
- Finally, Google Ads is a platform supporting advertising on the different Google services, so again it is not precisely a social media platform. We did not setup tracking conversions (filling and submitting the survey) but only the click on the advertisement, which means landing on the survey page.

We selected these different channels, and mainly social media, to share the link to the questionnaire survey in order to recruit the most extensive possible population panel. It is essential to highlight that the survey participants’ recruitment was done manually (by a human operator) by publishing the survey address without using a paid service using dedicated algorithms to optimize the distribution of the message. Consequently, we anticipated a low expected response rate. In view of the estimations of the response rate directly generated by the Survey questionnaire link over social media (Table 1), the possible response rate was over 0.5% (1728 participants out of a global estimated post reach size approximatively of 305,500). This value means that the response rate is a moderate one over all the social medias used. 

For spreading the survey over social media, we used personal accounts (not related to a dedicated page or to a business) and accordingly we were not able to get accurate data for each social media. Furthermore, some social media platforms were not delivering target and reach size estimates but only the number of users’ interactions with the post. Regarding the email campaign, we used a tracking system reporting the ‘first mail opening by each receiver and click on the survey’ link.

Participants were volunteers, unpaid, and anonymous; those over 18 years old were eligible to complete the survey. Each individual had to certify eligibility before answering the questionnaire and agree to participate in the survey.

The questionnaire included 12 closed questions distributed into three subsets, focusing on:

1. The intent and the reasons to take and recommend the COVID-19 vaccine;
2. The person’s health status regarding the COVID-19 disease;
3. Socio-demographics.

The survey was reviewed and approved by the ethics committee of the Faculty of Industrial Engineering and Management of the Holon Institute of Technology (TM/2/2020/AB/003). The information provided by the participants during the survey is stored in a secured, encrypted manner, with restricted access services provided by the principal researcher’s institution.

### 2.2. Data Preparation

Participants had to answer at least two mandatory questions related to COVID-19 vaccination adherence: “will you (or did you) take the COVID-19 vaccine?” and “will you recommend the COVID-19 immunization to your friends and family?” The other ten questions were optional. The answer set inclusion criteria were declaring a US state of residence and completing the overall questionnaire in more than one minute.

The manuscript adheres to reporting standards, including the Checklist for Reporting Results of Internet E-Surveys (CHERRIES) [21], the Strengthening the Reporting of Observational Studies in Epidemiology (STROBE) [22], and Transparent Reporting of a Multivariable Prediction Model for Individual Prognosis or Diagnosis (TRIPOD) guidelines for reporting observational studies [23].

### 2.3. Statistical Analysis

All answer sets that do not fit the inclusion criteria were removed. The comprehensive data collected in the context of this research were categorical.

Before we started the statistical analysis process, we reformulated part of the data. The question concerning the reasons for taking or not taking the COVID-19 vaccine was in the form of matrix point rating multiple-choice questions with a five-point Likert scale (“completely disagree”, “somewhat disagree”, “neutral/no opinion”, “somewhat agree”, and “completely agree”). For the statistical analysis, the “disagree” and “agree” answers were aggregated into these two categories.

Some socio-demographic answers were recoded to increase data consistency. Participants’ responses declaring an age of over 75 years were merged with those of the next-oldest group (65–74 years), producing a new group instead (≥65 years). For education level, the answers “less than high school” and “high school or some college” were merged as “no college degree”. The number of children was aggregated as follows: “0”, “1–2”, and “3 or more”. The participants’ states of residence were aggregated to nine divisions and four regions.

In the first step of the analysis, the collected data were stratified by answering the question, “will you (or did you) take the COVID-19 vaccine?” The distribution of the responses was presented as numbers and percentages. Chi-square tests and Fisher’s exact tests were used to compare the variables. 

Cronbach’s α was used to measure the internal reliability of the Likert scale question related to the reasons to take and recommend the COVID-19 vaccine (α = 0.76). Then, logistic regression was used to determine the association of the intent to get the COVID-19 vaccine with the participants’ socio-demographic data and reasons. In the first part of the analysis, although the three levels stratified the answers, we considered that the absence of a clear intent to get the COVID-19 vaccine must be handled as a risk of not taking it [24,25]. Therefore, the vaccination intent was dichotomized to “vaccination adherence” for “yes” and “vaccination hesitancy” for the hesitant (“maybe”) or opponent (“no”) individuals [26]. First, the crude odds ratios were calculated. Then, we computed two logistic regression models. Model 1 was adjusted with all the predictor variables (answers to the survey). Model 2 was adjusted to a subset of predictor variables selected by running stepwise logistic regression, reducing the model’s complexity without compromising its predictive accuracy.

Next, we took the predictors of model 2 and used them to build a decision tree [27] (model 3), allowing a much better understanding of the main factors involved in vaccination adherence or vaccination hesitancy. This third model’s objective was to overcome Simpson’s paradox [27,28] by displaying nonlinear interactions between predictors in an easily interpretable way. The last step of the analysis consisted of evaluating the models. 

The data analysis was performed with R and the “psych” package [29] for computing the answers’ internal consistency for the matrix multi-point scale questions, the “compareGroups” package [30] for statistical computations, and the “rpart” package [31] for the decision-tree processing. In the overall analysis, statistical significance was considered as a two-sided *p* ≤ 0.05.

## 3. Results

We collected 1728 answer sets. After applying the exclusion criteria (42 answered in less than one minute, and 42 did not have their state of residence), 1644 answer sets were included in the analysis. Completing the Survey questionnaire took a median time of 102.5 s (IQR (83.25;135.00)).

### 3.1. Socio-Demographic Characteristics

The participants had a border median age group of 35–44 (48.8% in the range 18–34 years), were mainly women (53.9%), married or in a civil union (56.7%), without children (55.4%), and had higher education (75.9%). Furthermore, they were white (83.0%) and lived in the North Central (33.3%) and South regions (30.0%) of the USA. Nevertheless, it is essential to highlight that the proportion of single individuals was relatively high (36.1%), and individuals with one to two children comprised 33.9% (Table 2). The median age in the US population was 38.2 years in 2020, roughly matching the survey population’s median age.

The participants answering that they intend to get the vaccine, as compared to those responding “maybe” or “no”, were younger (73.8% below 45 years old, with a median age of 25–34 years, vs. 66.71% below 45 years, with a median age of 35–44 years, respectively). Women were also dominant and strongly represented in the “maybe” group (60.3%). The proportion of the respondents having at least three children was higher for those answering “maybe” or “no” than for those in the general population of the survey (12.4% and 21.4% vs. 9.45%).

Another difference between the adherent and hesitant individuals was education level. The proportion of individuals without a college degree was higher in the “maybe” and “no” groups than in the “yes” group (26.9% and 26.1% vs. 22.5%, respectively). Additionally, ethnic minorities were more often represented in the “maybe” and “no” groups than in the adherent group (19.27% and 19.7% vs. 14.38%, respectively). Moreover, the proportion declining to declare their ethnicity was higher in those groups (4.83% and 10.2% vs. 1.72%, respectively) as well. The geographical division (*p* = 0.708) or region (*p* = 0.486) of residence did not influence the decision to be vaccinated. The health status regarding COVID-19 of participants or their relatives (Table 3), defined as high risk for COVID-19 complications, was higher in the “yes” group (70.2%) than in both “maybe” and “no” groups (64.1% and 60.1%, respectively). However, the proportion of participants or their relatives positively diagnosed with COVID-19 was lower among the participants who answered that they would take the vaccine (31.0% vs. 36.6% and 43%, respectively).

Focusing specifically on the younger segment of the participants in the survey (aged 18–34 years) demonstrated the importance of ethnicity: a higher vaccination hesitancy was noted among minorities (21.57%) than among the white population (14.51%, *p* < 0.001). No significant differences were observed in this group regarding other socio-demographic attributes.

### 3.2. Participants’ Reasons for Taking the COVID-19 Vaccine

**Figure 2:**
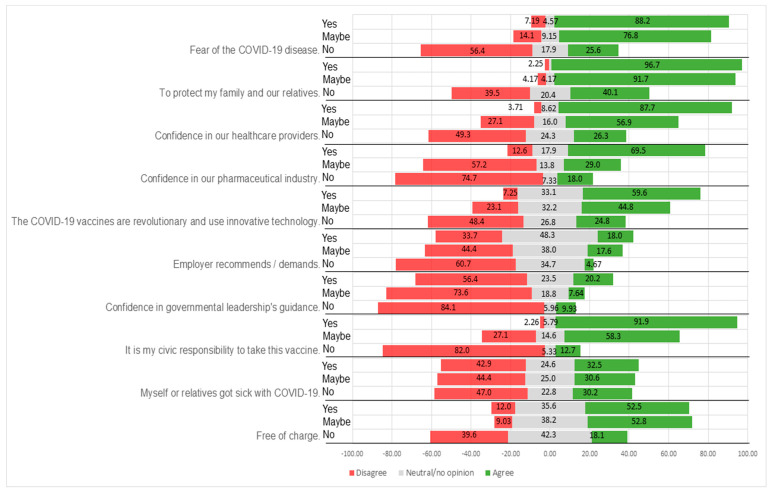
Likert plot of the reasons of the participants in the survey for taking or not taking the COVID-19 vaccine.

The survey participants were asked to report their agreement or disagreement with reasons for taking the COVID-19 vaccine (Figure 2, Table 4). Thus, individuals declaring vaccination adherence or hesitancy both specified the reasons that motivated them. The survey participants who answered that they would take the vaccine justified their decision by the need to protect family and relatives (96.7% of the group), as a civic responsibility (91.9%), and out of their worry regarding COVID-19 (88.2%). They also had high confidence levels in the healthcare system (87.7% in healthcare providers and 69.5% in the pharmaceutical industry). However, 17.9% were neutral, which could reflect some lack of trust in vaccine producers.

Likewise, individuals declaring that they may take the vaccine indicated a need to protect family and relatives (91.7%) and worry about the disease (76.8%). This population segment had not yet made a clear decision. Indeed, this group showed some ambivalence regarding vaccination as a civic responsibility (58.3%) and evinced lower confidence in healthcare providers (56.9%) and the pharmaceutical industry (57.2%).

For both groups above, the innovative technologies used in developing the COVID-19 vaccines [32] and that they will be free of charge motivated their adherence (52.5%.

The vaccine opponents strongly disagreed that being vaccinated against COVID-19 was a civic responsibility (82.0%), and they lack confidence in the pharmaceutical industry (74.4%) and, to some extent, in healthcare providers as well (49.3%). Moreover, fear of the disease did not justify vaccination for 56.4% of the group’s individuals. Protection of family and relatives was well balanced between agreement (40.1%) and disagreement (39.5%). This presumably means that those who agree think that not being vaccinated helps keep relatives safe in this group’s specific context. Those who disagree are not taking the vaccine for the same reason.

For the overall survey population, vaccinating against COVID-19 did not appear to depend on an employer recommendation or demand, as 83.2% of participants were neutral or disagreed that this was a factor. This may reflect the vaccines’ novelty, as it would have been difficult for an employer to mandate vaccination before the vaccines were widely available. From another perspective, the low level of trust in government leadership (18.1%) does not look like a factor explaining the decision to take or not take the vaccine.

We noticed that an intent to take the vaccine was correlated in 96.8% of the participants with a recommendation for others to vaccinate as well. The undecided respondents were also undecided regarding recommending vaccination (64.8%). Again, opponents mostly would not recommend vaccination (75.5%); however, 6.9% of these nevertheless would recommend vaccination.

The younger groups in this survey (aged 18–34 years) showed highly significant differences regarding the reasons to vaccinate or not vaccinate between the adherent and the hesitant (*p* < 0.001). Moreover, the proportion of adherents recommending vaccination to others was slightly higher than in the overall population (97.5% vs. 96.8%), but with a similar behavior characterizing the opponents not recommending vaccination (78%).

All the results disclosed above were significant with *p* < 0.05. It should be highlighted that the fact that the participant or one of his/her relatives was sick with COVID-19 was not considered a reason for taking the vaccine (*p* = 0.897).

### 3.3. Multivariate Analysis

Model 1 enabled us to consider the systemic aspects of vaccination adherence by looking at each one of the participants as a whole, and at the person’s socio-demographic details, health status, and sentiments concerning COVID-19 vaccination as interdependent variables [33]. Model 2 was built around a limited number of factors selected as the most influential.

Socio-demographic factors were correlated with the decision to get the vaccine. Even in the crude data, middle-aged participants (age group 45–54 years) were most likely to intend to get the vaccine (age group 45–54 years OR = 1.95 (95% CI 1.14–3.41), and age group 55–64 years, OR = 1.26 (95% CI 0.65–2.45, *p* = 0.044)). The adjusted models showed that young individuals (aged 18–24 years) were also highly motivated for immunization (respectively, in model 1 OR = 2.32 (95% CI 1.01–5.63, *p* = 0.053) and in model 2 OR = 2.04 (95% CI 0.99–4.63, *p* = 0.053)).

In both crude and adjusted models, males were less intent on taking the vaccine (OR < 0.80 (95% CI 0.25–1.04, *p* = 0.003)) than females and individuals not defining their gender. Additionally, the greater the number of children was, the greater the likelihood of planning to get the COVID-19 vaccine (*p* < 0.0001).

Some of the reasons for being vaccinated were common in both models, such as fear of the disease (OR = 1.90 (95% CI 1.01–3.50, *p* < 0.0001)), protection of family and relatives (OR = 0.90 (95% CI 0.36–2.19, *p* < 0.0001)), confidence in healthcare providers and the pharmaceutical industry (OR = 2.81 (95% CI 1.50–5.29), *p* < 0.0001), and 6.83 (95% CI 4.23–11.13, *p* < 0.0001), respectively), and the feeling that getting the vaccine is a civic responsibility (OR = 32.39 (95% CI 17.63–61.86, *p* < 0.0001)).

**Figure 3:**
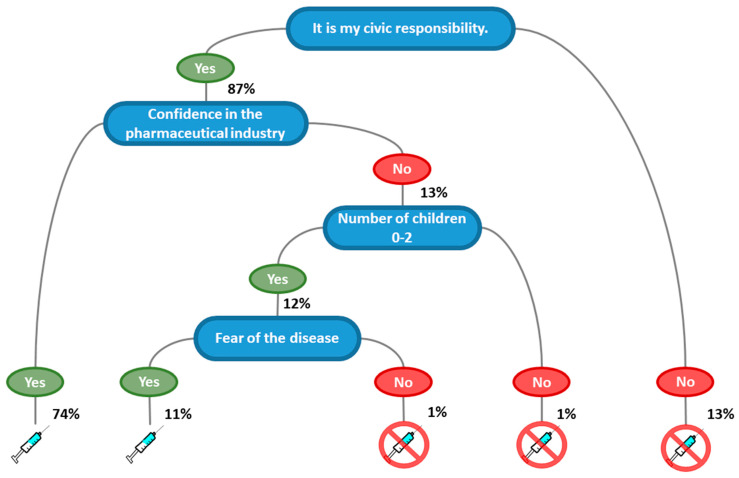
Decision tree for taking the COVID-19 vaccine based on stepwise logistic regression.

Based on the factors in model 2, we built a regression decision tree (model 3) to support health communication specialists in targeting their efforts to increase vaccination adherence (Figure 3). The most crucial factor determining adherence or hesitancy was the concept that getting the vaccine constituted a civic responsibility, followed by confidence in the pharmaceutical industry (74% of vaccination adherents). Not considering vaccination as a civic responsibility was correlated with vaccination hesitancy (13%). Moreover, feeling responsible even if lacking confidence in the pharmaceutical industry led to a positive intent if the participants had fewer than three children and worried about COVID-19 (11%). Feeling responsibility but not being confident in the pharmaceutical industry led to vaccination hesitancy for those with more than two children or who were neutral regarding worry about the disease (2%).

We proceeded to predict the performance of models 1, 2, and 3 (Table 5) by having the models learn on 90% of the answer sets and then tested the learned models on the remaining 10%. Model 1 (fully adjusted) enabled predicting the intent to get the vaccine with an accuracy of 82.25%, model 2 with an accuracy of 84.84%, and model 3 with an accuracy of 87.45%.

Vaccination adherence is mainly affected by individual opinions and health literacy, which is potentially reflected in this survey by the participants’ age (median age group: 35–44), level of education (higher education: 75.9%), confidence in the healthcare providers (79.2%), and interpretations of civic responsibility (81.6%). Health communication on social media must consider both the audience’s interests and socio-demographic attributes [32].

Surprisingly, the decision-tree approach shows that the participants’ age group is not a major discriminator in COVID-19 vaccination adherence. Namely, individuals in all age groups in the survey have similar intent-to-adhere rates. However, from a policymakers’ perspective, young people (49.02% of participants below 35 years of age vs. 22% of the overall US population between 20 and 34 years old) intend to be vaccinated (83.3%) as a civic responsibility (83.3%) even if they are not defined as a priority group in the COVID-19 vaccination policies. This fits with prior research showing that prosocial motivations [31], such as the positive impact of vaccination on protecting relatives and community, more strongly predict the intent to vaccinate than self-protection [34,35]

## 4. Discussion

### 4.1. Principal Findings

To our knowledge, our study is the first on COVID-19 vaccination adherence and hesitancy that looked at the reasons disclosed by 1644 social media users without using established survey services. In contrast to prior research, we conducted our survey during the overlapping second and third waves, in parallel with approvals of the first two COVID-19 vaccines by the FDA and other agencies [8].

We have four major important findings:

1. Differences in opinions regarding civic responsibility are critical factors in vaccine acceptance (87%);
2. Confidence in the pharmaceutical industry (74%) has a positive impact on vaccination adherence;
3. The greater the number of children the participants had, the greater was their intent to get the COVID-19 vaccine (Table 4). Yet vaccination hesitancy exists (1%) in large families with no confidence in the pharmaceutical industry (Figure 2);
4. Fear of COVID-19 is a factor in determining the intent to be vaccinated. Naturally, worry increases adherence to vaccination against the disease (11%).

### 4.2. Strengths and Limitations

Running a survey on social media is a methodological limitation due to the passive exclusion of non-users or non-active users of online social media, but the widespread use of social media may allay such concerns. After all, approximately 90% of the US population aged 18–29 uses social media [36]. Furthermore, the potential risk of having non-US residents answering exists, even if one key factor is having some high degree of trust in the participants. Also, running this kind of survey dissemination in a “manual” manner without using automated and paying systems requires defining some targets having a potential response rate due to their focus. 

Targeting the recruitment of survey participants to groups focusing on a limited list of subjects necessarily introduces some sort of bias. Indeed, the members of such communities of interest may have backgrounds that increase their compliance or hesitancy with regard to vaccination. Additionally, it is essential to point out that some group moderators removed our “call-to-participate” message. They justified their decision by not agreeing to involve their group members in a survey, with a few explicitly declining to involve their members in a study encouraging vaccination, according to their understanding. These refusals induce a bias similar to real-world behavior wherein vaccine opponents are restraining their communities’ involvement in this kind of research.

Moreover, we observed that some individuals, in some social media groups, expressed a fear of being tracked by answering our questionnaire. It is essential to bear in mind that vaccination hesitancy includes individuals who have not yet rejected vaccination but who distrust institutions or companies related to vaccination. This distrust may extend to researchers analyzing vaccination-related behavior [37].

Our study shows that developing models predicting vaccine adherence or hesitancy based on easy-to-collect data not related to a specific disease or treatment delivers high-quality outputs (around 85%). Our predictive machine-learning models can support health communication policies to improve the dissemination and the impact of evidence-based information on vaccines and vaccination [38,39].

We have demonstrated that using social media to survey non-specifically targeted participants highlights that young individuals intend to be vaccinated in the USA, even if they are not defined as a priority group according to the current COVID-19 vaccination policies. Moreover, this is in contrast with the traditional media reporting high vaccination hesitancy in the young population. Furthermore, we point out that the intent to be vaccinated resides in personal convictions, such as feelings of civic responsibility and confidence in the healthcare and pharmaceutical industries, more than socio-demographic variables. 

As of March 2021, many countries are experiencing their third wave of the COVID-19 pandemic, parallel with approved vaccines against the disease. Therefore, the acceptance of these vaccines to control the pandemic is of prime public health importance. Our study shows that increasing vaccination rates and reducing hesitancy require a communication approach leading to citizen engagement, associated with enhancing health literacy. It is vital to increase confidence in the healthcare system and industry by eliminating myths and rumors. The variables related to the intent to vaccinate found in this study appear generalizable and could have worldwide benefits [40].

## 5. Conclusions

Vaccination adherence and hesitancy against COVID-19 are mainly affected by individual opinions and health literacy.

### 5.1. Implications for Healthcare Practice

Healthcare policymakers must consider communication on social media as a strategic task. Therefore, they should contemplate making use of social media advertising campaigns. They must target the vaccination hesitancy groups (18.5% or 304/1644 in this study) to stimulate the civic motivation for vaccination (62.8% or 191/304 of the hesitant participants denying this reason) and to counter mistrust in healthcare providers (57.2% or 174/304) and the pharmaceutical industry (74.3% or 226/304). Additional campaigns should target the vaccination adherence group (81.5% or 1340/1644) as a retention program and reminder to act. Public health communication with a specific audience must employ adapted and personalized language that considers personal opinions and fears [41] and addresses concerns about the risks and benefits of the COVID-19 vaccine [42,43].

Improving adherence to COVID-19 vaccination must start, in this era of social media-based knowledge, by building trust [26]. Healthcare policymakers should use community influencers to effectively promulgate trustworthy vaccination messages and dismantle dangerous false claims [42,44]. This approach should have a powerful impact on about 10% (23.5 million people [36]) of the overall US population using social media (recall that 8.9% of this study’s participants declare that they may take the COVID-19 vaccine). Policymakers can also use our results in their reinforcement advertising to publicly reassure younger people that they have done the right thing in opting for vaccination [45].

### 5.2. Current and Potential Future Research Directions

The overall population is becoming more and more accustomed to using social media to get information and enhance their knowledge [46]. Analyzing, on a few specific social media platforms, concerns voiced about COVID-19 vaccination (or any other vaccine) can help policymakers to understand how other aspects of daily life are considered related to it in specific population segments. This understanding can help to improve the targeting of communication campaigns aimed at increasing vaccination compliance or at least professional and scientifically based tools for taking the vaccination decision. Additionally, the content of the communication campaign must use personalized content such as buzzwords, and pictures fitting with the target population specificities and non-vaccination related interests.

Moreover, the diversity of the US population shows the importance of running multilingual surveys to derive the knowledge needed to fine-tune communication about the importance of vaccination. Will a lower intent to adhere still be seen and still be associated with the same reasons for some minorities if the surveys are delivered in the appropriate language?

## Figures and Tables

**Table 1:** Estimations of the response rate directly generated by the Survey questionnaire link over social media.

Social Media	Estimated Post Reach Size ^1,2^	Estimated Responses ^3^	Possible Response Rate (%) ^5^
Facebook	200,000	400	0.20
Reedit	100,000	700	0.70
Twitter	1248	68	5.45
Instagram	100	5	5.00
LinkedIn	1000	25	2.50
Email	2115 ^4^	150 ^4^	7.09
Google Ads	1067	12	1.12

^1^ Estimated post reach sizes consider the reported views of the survey advertising post for each social media over the different targeted group of following threads related to subjects of interest (such as COVID-19 or health). ^2^ When accurate data were not available, we considered that the post reach size was, in the better case, the third of the overall possible target (i.e., Facebook, Reedit, Instagram). ^3^ Responses include likes or votes, comments or replies, and sharing. ^4^ Estimated reach is based on the reports generated by an email tracking system (first mail opening by each receiver and first click on the survey’ link). ^5^ Ratio between the “estimated responses” and the “estimated post reach size”.

**Table 2:** Socio-demographic characteristics of the participants in the survey.

	Overall (*n* = 1644)	Yes (*n* = 1340)	Maybe (*n* = 145)	No (*n* = 159)	*p*-Value
**Age group (*n* = 1643)**					
18–24	163 (9.92%)	137 (10.2%)	12 (8.28%)	14 (8.81%)	
25–34	643 (39.1%)	534 (39.9%)	64 (44.1%)	45 (28.3%)	
35–44	445 (27.1%)	367 (27.4%)	31 (21.4%)	47 (29.6%)	
45–54	218 (13.3%)	159 (11.9%)	24 (16.6%)	35 (22.0%)	
55–64	119 (7.24%)	96 (7.17%)	10 (6.90%)	13 (8.18%)	
65 and more	47 (2.86%)	39 (2.91%)	4 (2.76%)	4 (2.52%)	
Prefer to not say	8 (0.49%)	7 (0.52%)	0 (0.00%)	1 (0.63%)	
**Gender (*n* = 1642)**					0.016
Female	885 (53.9%)	711 (53.1%)	91 (62.8%)	83 (52.2%)	
Male	716 (43.6%)	599 (44.8%)	47 (32.4%)	70 (44.0%)	
Non-binary	30 (1.83%)	23 (1.72%)	4 (2.76%)	3 (1.89%)	
Prefer not to say	11 (0.67%)	5 (0.37%)	3 (2.07%)	3 (1.89%)	
**Marital status (*n* = 1642)**					0.005
Single	592 (36.1%)	492 (36.8%)	53 (36.6%)	47 (29.6%)	
Married/civil union	931 (56.7%)	763 (57.0%)	78 (53.8%)	90 (56.6%)	
Separated/divorced	89 (5.42%)	64 (4.78%)	7 (4.83%)	18 (11.3%)	
Widowed	13 (0.79%)	10 (0.75%)	2 (1.38%)	1 (0.63%)	
Prefer not to say	17 (1.04%)	9 (0.67%)	5 (3.45%)	3 (1.89%)	
**Children (*n* = 1641)**					<0.001
0	909 (55.4%)	772 (57.7%)	73 (50.3%)	64 (40.3%)	
1–2	556 (33.9%)	455 (34.0%)	48 (33.1%)	53 (33.3%)	
3 and more	155 (9.45%)	103 (7.70%)	18 (12.4%)	34 (21.4%)	
Prefer to not say	21 (1.28%)	7 (0.52%)	6 (4.14%)	8 (5.03%)	
**Education level (*n* = 1635)**					0.018
No college degree	380 (23.2%)	300 (22.5%)	39 (26.9%)	41 (26.1%)	
Bachelor’s degree	652 (39.9%)	528 (39.6%)	56 (38.6%)	68 (43.3%)	
Postgraduate degree	589 (36.0%)	498 (37.4%)	47 (32.4%)	44 (28.0%)	
Prefer to not say	14 (0.86%)	7 (0.53%)	3 (2.07%)	4 (2.55%)	
**Ethnicity (*n* = 1639)**					<0.001
White	1342 (81.9%)	1122 (83.9%)	110 (75.9%)	110 (70.1%)	
American Indian and Alaska Native	14 (0.85%)	11 (0.82%)	0 (0.00%)	3 (1.91%)	
Asian	101 (6.16%)	84 (6.28%)	12 (8.28%)	5 (3.18%)	
Black or African American	29 (1.77%)	17 (1.27%)	2 (1.38%)	10 (6.37%)	
Hispanic or Latino	60 (3.66%)	44 (3.29%)	8 (5.52%)	8 (5.10%)	
Native Hawaiian and other Pacific Islander	2 (0.12%)	2 (0.15%)	0 (0.00%)	0 (0.00%)	
Two or more races	45 (2.75%)	34 (2.54%)	6 (4.14%)	5 (3.18%)	
Prefer not to say	46 (2.81%)	23 (1.72%)	7 (4.83%)	16 (10.2%)	
**Residence by division (*n* = 1644)**					0.708
East North Central	461 (28.0%)	370 (27.6%)	44 (30.3%)	47 (29.6%)	
East South Central	80 (4.87%)	68 (5.07%)	7 (4.83%)	5 (3.14%)	
Middle Atlantic	185 (11.3%)	152 (11.3%)	14 (9.66%)	19 (11.9%)	
Mountain	139 (8.45%)	105 (7.84%)	16 (11.0%)	18 (11.3%)	
New England	70 (4.26%)	55 (4.10%)	8 (5.52%)	7 (4.40%)	
Pacific	208 (12.7%)	169 (12.6%)	23 (15.9%)	16 (10.1%)	
South Atlantic	247 (15.0%)	206 (15.4%)	19 (13.1%)	22 (13.8%)	
West North Central	87 (5.29%)	73 (5.45%)	4 (2.76%)	10 (6.29%)	
West South Central	167 (10.2%)	142 (10.6%)	10 (6.90%)	15 (9.43%)	
**Residence by region (*n* = 1644)**					0.486
North Central	548 (33.3%)	443 (33.1%)	48 (33.1%)	57 (35.8%)	
Northeast	255 (15.5%)	207 (15.4%)	22 (15.2%)	26 (16.4%)	
South	494 (30.0%)	416 (31.0%)	36 (24.8%)	42 (26.4%)	
West	347 (21.1%)	274 (20.4%)	39 (26.9%)	34 (21.4%)	

**Table 3:** Health status characteristics regarding COVID-19 of the participants in the survey.

	Overall (*n* = 1644)	Yes (*n* = 1340)	Maybe (*n* = 145)	No (*n* = 159)	*p*-Value
**Participant or relatives defined as high risk for COVID-19 complications (*n* = 1643)**					0.002
No	482 (29.3%)	376 (28.1%)	51 (35.2%)	55 (34.8%)	
Prefer to not say	30 (1.83%)	17 (1.27%)	5 (3.45%)	8 (5.06%)	
Yes	1131 (68.8%)	947 (70.7%)	89 (61.4%)	95 (60.1%)	
**Participant or relatives positively diagnosed with COVID-19 (*n* = 1640)**					0.005
No	1105 (67.4%)	923 (69.0%)	92 (63.4%)	90 (57.0%)	
Yes	535 (32.6%)	414 (31.0%)	53 (36.6%)	68 (43.0%)	

**Table 4:** Reasons for taking, or not taking, the COVID-19 vaccine as stated by the participants in the survey.

	Overall (*n* = 1644)	Yes (*n* = 1340)	Maybe (*n* = 145)	No (*n* = 159)	*p*-Value
**Will you recommend the COVID-19 immunization to your friends and family? (*n* = 1644)**					0.001
Yes	1342 (81.6%)	1297 (96.8%)	34 (23.4%)	11 (6.92%)	
Maybe	161 (9.79%)	39 (2.91%)	94 (64.8%)	28 (17.6%)	
No	141 (8.58%)	4 (0.30%)	17 (11.7%)	120 (75.5%)	
**Fear of the COVID-19 disease (*n* = 1633)**					<0.001
Agree	1327 (81.3%)	1178 (88.2%)	109 (76.8%)	40 (25.6%)	
Neutral/no opinion	102 (6.25%)	61 (4.57%)	13 (9.15%)	28 (17.9%)	
Disagree	204 (12.5%)	96 (7.19%)	20 (14.1%)	88 (56.4%)	
**To protect my family and our relatives (*n* = 1630)**					<0.001
Agree	1483 (91.0%)	1290 (96.7%)	132 (91.7%)	61 (40.1%)	
Neutral/no opinion	51 (3.13%)	14 (1.05%)	6 (4.17%)	31 (20.4%)	
Disagree	96 (5.89%)	30 (2.25%)	6 (4.17%)	60 (39.5%)	
**Confidence in** **healthcare providers (*n* = 1618)**					<0.001
Agree	1281 (79.2%)	1159 (87.7%)	82 (56.9%)	40 (26.3%)	
Neutral/no opinion	174 (10.8%)	114 (8.62%)	23 (16.0%)	37 (24.3%)	
Disagree	163 (10.1%)	49 (3.71%)	39 (27.1%)	75 (49.3%)	
**Confidence in the pharmaceutical industry (*n* = 1619)**					<0.001
Agree	989 (61.1%)	920 (69.5%)	42 (29.0%)	27 (18.0%)	
Neutral/no opinion	268 (16.6%)	237 (17.9%)	20 (13.8%)	11 (7.33%)	
Disagree	362 (22.4%)	167 (12.6%)	83 (57.2%)	112 (74.7%)	
**The COVID-19 vaccines are revolutionary and use innovative technology (*n* = 1621)**					<0.001
Agree	892 (55.0%)	790 (59.6%)	64 (44.8%)	38 (24.8%)	
Neutral/no opinion	526 (32.4%)	439 (33.1%)	46 (32.2%)	41 (26.8%)	
Disagree	203 (12.5%)	96 (7.25%)	33 (23.1%)	74 (48.4%)	
**Employer recommends/demands (*n* = 1613)**					<0.001
Agree	270 (16.7%)	238 (18.0%)	25 (17.6%)	7 (4.67%)	
Neutral/no opinion	744 (46.1%)	638 (48.3%)	54 (38.0%)	52 (34.7%)	
Disagree	599 (37.1%)	445 (33.7%)	63 (44.4%)	91 (60.7%)	
**Confidence in government leadership’s guidance (*n* = 1620)**					<0.001
Agree	293 (18.1%)	267 (20.2%)	11 (7.64%)	15 (9.93%)	
Neutral/no opinion	347 (21.4%)	311 (23.5%)	27 (18.8%)	9 (5.96%)	
Disagree	980 (60.5%)	747 (56.4%)	106 (73.6%)	127 (84.1%)	
**It is my civic responsibility to take this vaccine (*n* = 1623)**					<0.001
Agree	1325 (81.6%)	1222 (91.9%)	84 (58.3%)	19 (12.7%)	
Neutral/no opinion	106 (6.53%)	77 (5.79%)	21 (14.6%)	8 (5.33%)	
Disagree	192 (11.8%)	30 (2.26%)	39 (27.1%)	123 (82.0%)	
**Myself or relatives got sick with COVID-19 (*n* = 1617)**					0.897
Agree	519 (32.1%)	430 (32.5%)	44 (30.6%)	45 (30.2%)	
Neutral/no opinion	396 (24.5%)	326 (24.6%)	36 (25.0%)	34 (22.8%)	
Disagree	702 (43.4%)	568 (42.9%)	64 (44.4%)	70 (47.0%)	
**Free of charge (*n* = 1606)**					<0.001
Agree	792 (49.3%)	689 (52.5%)	76 (52.8%)	27 (18.1%)	
Neutral/no opinion	585 (36.4%)	467 (35.6%)	55 (38.2%)	63 (42.3%)	
Disagree	229 (14.3%)	157 (12.0%)	13 (9.03%)	59 (39.6%)	

**Table 5:** Univariate crude and adjusted logistic regression models for odds ratio of intent to take the COVID-19 vaccine.

	Crude OR (95% CI)	*p*-Value	Model 1 OR (95% CI)	*p*-Value	Model 2 OR (95% CI)	*p*-Value
**Age group (*n* = 1643)**						
18–24	1.00 (--)	0.044	1.00 (--)	0.053	1.00 (--)	0.053
25–34	1.08 (0.66;1.79)		2.32 (1.01;5.63)		2.04 (0.99;4.63)	
35–44	1.12 (0.68;1.90)		1.30 (0.50;3.48)		1.23 (0.52;3.01)	
45–54	1.95 (1.14;3.41)		2.71 (0.98;7.71)		2.18 (0.88;5.58)	
55–64	1.26 (0.65;2.45)		1.17 (0.34;4.03)		0.98 (0.31;3.03)	
65 and more	1.08 (0.39;2.71)		2.87 (0.37;16.28)		1.67 (0.28;8.20)	
Prefer to not say	0.75 (0.02;6.28)		0.01 (0.00;0.57)		0.03 (0.00;1.05)	
**Gender (*n* = 1642)**						
Female	1.00 (--)	0.006	1.00 (--)	0.003	1.00 (--)	0.003
Male	0.80 (0.61;1.04)		0.39 (0.25;0.62)		0.43 (0.27;0.65)	
Non-binary	1.24 (0.44;3.05)		1.12 (0.28;4.25)		1.03 (0.22;3.67)	
Prefer not to say	4.89 (1.23;20.51)		2.49 (0.08;52.64)		5.32 (0.38;51.13)	
**Status (*n* = 1642)**						
Single	1.00 (--)	0.003	1.00 (--)	0.552		
Married/civil union	1.08 (0.82;1.44)		1.09 (0.61;1.94)			
Separated/divorced	1.92 (1.10;3.27)		1.10 (0.40;2.89)			
Widowed	1.47 (0.26;5.87)		0.21 (0.01;2.68)			
Prefer not to say	4.36 (1.43;13.08)		2.16 (0.17;18.68)			
**Children (*n* = 1641)**						
0	1.00 (--)	<0.0001	1.00 (--)	<0.001	1.00 (--)	<0.0001
1–2	1.25 (0.93;1.67)		1.51 (0.87;2.65)		1.56 (0.96;2.53)	
3 and more	2.84 (1.90;4.22)		3.88 (1.80;8.37)		3.80 (1.89;7.56)	
Prefer to not say	11.22 (4.15;33.45)		8.76 (1.34;49.11)		10.96 (2.05;51.19)	
**Education (*n* = 1635)**						
No college degree	1.00 (--)	0.001	1.00 (--)	0.06		
Bachelor’s degree	0.88 (0.64;1.22)		1.00 (0.59;1.70)			
Postgraduate degree	0.69 (0.48;0.97)		0.79 (0.45;1.39)			
Prefer to not say	3.73 (1.08;12.88)		3.30 (0.14;40.97)			
**Ethnicity (*n* = 1639)**						
White	1.00 (--)	<0.0001	1.00 (--)	<0.001		
American Indian/Alaska Native	1.39 (0.25;5.32)		0.26 (0.01;4.91)			
Asian	1.03 (0.56;1.80)		0.91 (0.33;2.32)			
Black or African American	3.60 (1.54;8.12)		0.21 (0.04;1.24)			
Hispanic or Latino	1.85 (0.96;3.42)		2.39 (0.88;5.97)			
Native Hawaiian/Pacific Islander	0.00 (0.00;27.25)		-			
Prefer not to say	5.09 (2.68;9.68)		1.29 (0.33;4.95)			
Two or more races	1.65 (0.74;3.40)		1.76 (0.58;4.74)			
**Residence by region (*n* = 1644)**						
North Central	1.00 (--)	0.254	1.00 (--)	0.129		
Northeast	0.98 (0.65;1.45)		0.76 (0.39;1.44)			
South	0.79 (0.56;1.10)		0.64 (0.39;1.06)			
West	1.12 (0.79;1.59)		0.81 (0.46;1.40)			
**Participant or relatives defined as high-risk of COVID-19 complications (*n* = 1643)**						
No	1.00 (--)	<0.001	1.00 (--)	0.004		
Prefer to not say	2.71 (1.17;6.13)		2.31 (0.45;10.26)			
Yes	0.69 (0.52;0.91)		1.06 (0.68;1.68)			
**Participant or relatives positively diagnosed with** **COVID-19 (*n* = 1640)**						
No	1.00 (--)	0.003	1.00 (--)	0.020		
Yes	1.48 (1.13;1.93)		1.46 (0.86;2.47)			
**Fear of the COVID-19 disease** **(*n* = 1633)**						
Agree	1.00 (--)	<0.001	1.00 (--)	<0.0001	1 (--)	<0.0001
Neutral/no opinion	5.30 (3.35;8.33)		2.68 (1.25;5.60)		2.81 (1.34;5.72)	
Disagree	8.88 (6.35;12.44)		1.88 (0.96;3.58)		1.90 (1.01;3.50)	
**To protect my family and our relatives (*n* = 1630)**						
Agree	1.00 (--)	<0.001	1.00 (--)	<0.0001	1.00 (--)	<0.0001
Neutral/no opinion	17.60 (9.10;35.95)		4.71 (1.43;15.10)		4.08 (1.31;12.41)	
Disagree	14.66 (9.12;24.04)		1.88 (0.96;3.58)		0.90 (0.36;2.19)	
**Confidence in** **healthcare providers (*n* = 1619)**						
Agree	1.00 (--)	<0.001	1.00 (--)	<0.0001	1.00 (--)	<0.0001
Neutral/no opinion	4.99 (3.40;7.29)		1.08 (0.56;2.05)		1.11 (0.60;2.03)	
Disagree	22.01 (14.82;33.09)		2.83 (1.45;5.52)		2.81 (1.50;5.29)	
**Confidence in the pharmaceutical industry (*n* = 1619)**						
Agree	1.00 (--)	<0.001	1.00 (--)	<0.0001	1.00 (--)	<0.0001
Neutral/no opinion	1.74 (1.08;2.77)		1.36 (0.70;2.54)		1.25 (0.67;2.27)	
Disagree	15.52 (11.17;21.75)		7.66 (4.48;13.22)		6.83 (4.21;11.13)	
**The COVID-19 vaccines are revolutionary and use innovative technology (*n* = 1621)**						
Agree	1.00 (--)	<0.001	1.00 (--)	0.078		
Neutral/no opinion	1.53 (1.11;2.11)		0.71 (0.44;1.14)			
Disagree	8.61 (6.02;12.35)		1.04 (0.54;1.95)			
**Employer recommends/demands (*n* = 1613)**						
Agree	1.00 (--)	<0.001	1 (--)	0.859		
Neutral/no opinion	1.24 (0.80;1.95)		1.03 (0.57;1.91)			
Disagree	2.57 (1.69;4.02)		1.01 (0.55;1.89)			
**Confidence in government leadership’s guidance (*n* = 1620)**						
Agree	1.00 (--)	<0.001	1.00 (--)	0.864		
Neutral/no opinion	1.19 (0.68;2.11)		1.29 (0.61;2.80)			
Disagree	3.20 (2.07;5.12)		1.08 (0.57;2.15)			
**It is my civic responsibility to take this vaccine (*n* = 1623)**						
Agree	1.00 (--)	<0.001	1.00 (--)	<0.0001	1.00 (--)	<0.0001
Neutral/no opinion	4.46 (2.68;7.29)		2.94 (1.54;5.48)		3.04 (1.65;5.48)	
Disagree	63.64 (40.62;102.48)		36.59 (18.93;74.03)		32.39 (17.63;61.86)	
**Myself or relatives got sick with COVID-19 (*n* = 1617)**				0.655		
Agree	1.00 (--)	0.662	1.00 (--)			
Neutral/no opinion	1.04 (0.72;1.48)		1.32 (0.70;2.45)			
Disagree	1.14 (0.84;1.55)		1.26 (0.70;2.25)			
**Free of charge (*n* = 1606)**						
Agree	1.00 (--)	<0.001	1.00 (--)	0.725		
Neutral/no opinion	1.69 (1.25;2.28)		1.14 (0.72;1.79)			
Disagree	3.06 (2.13;4.40)		0.88 (0.42;1.78)			

## Data Availability

The data that support the findings of this study are available from the corresponding author (AB) upon reasonable request, which will need to undergo ethical and legal approvals by the institutions of the investigators of the research.

## Supplementary Materials

The following are available online at https://www.mdpi.com/article/10.3390/vaccines9040315/s1, Supplementary material S1: Survey questionnaire.
