# Supplementary material for: Reasons for Taking the COVID-19 Vaccine by US Social Media Users
Source: Vaccines (Basel). 2021 Mar 29;9(4):315. doi: 10.3390/vaccines9040315 (PMC8067223; doi:10.3390/vaccines9040315)
Supplement: Supplementary file 1 [file vaccines-09-00315-s001.pdf]

Supplementary material: Survey questionnaire

Dear Participant,

With the rise of public interest and concerns around the new COVID-19 vaccines, it is important for our Public Health scientific community to understand which factors influence vaccination compliance. To this end, we have prepared a short survey, where you can anonymously express your opinion about the COVID-19 vaccination.

This survey focuses on the United States of America only. Please fill it only if you are living in the USA. The survey should guide the decision-makers to adapt their public health strategy.

We will be grateful if you will dedicate, in a voluntary and anonymous manner, five minutes to address the following 12 questions.

* At the end of the questionnaire, you need to submit it by clicking on "Submit".

Thanks for taking a part in this survey.

Stay safe,

The Investigators

Prof. Abraham Seidmann, Boston University, Boston, MA, USA; Dr. Arriel Benis, Holon Institute of Technology, Holon, Israel; Prof. Shai Ashkenazi, Ariel University, Israel

This survey has been granted by the relevant Ethics Committee of the Holon Institute of Technology (https://www.hit.ac.il/en/).

By moving to the next page of this survey, I hereby certify that I am over eighteen (18) years of age and I agree to take part in it.

1. Will you recommend the COVID-19 immunization to your friends and family?
    - Yes
    - No
    - Maybe
2. Will you (or did you) take the COVID-19 vaccine?
    - Yes
    - No
    - Maybe

3. Please explain your motivation to vaccinate or not

	Completely disagree	Somewhat disagree	Neutral/ No opinion	Somewhat agree	Completely agree
Fear of the COVID-19 disease					
To protect my family and our relatives					
Confidence in our healthcare providers					
Confidence in our pharmaceutical industry					
The COVID-19 vaccines are revolutionary and use innovative technology					
Employer recommends / demands					
Confidence in governmental leadership's guidance					
It is my civic responsibility to take this vaccine					
Myself or relatives got sick with COVID-19					
Free of charge					

4. Are you, or any close members of your family, at high risk of COVID-19 complications (due to Obesity, Cardiovascular disease, Respiratory disease, or any other chronic disease)?
    - Yes
    - No
    - Prefer to not say
5. Were you, or one of your relatives, positively diagnosed with COVID-19?
    - Yes
    - No
6. What is your age group (in years)?
    - 18-24
    - 25-34
    - 35-44
    - 45-54
    - 55-64
    - 65-74
    - 75+
7. What is your gender?
    - Woman
    - Man
    - Non-binary
    - Prefer to not say
    - Other
8. What is your marital status?
    - Single
    - Married / Civil Union
    - Separated / Divorced

- Widowed
- Prefer not to say

9. How many children do you have?
   - 0
   - 1
   - 2
   - 3
   - 4 and more
10. What is your education level?
    - Less than high school
    - High school or some college
    - Bachelor's degree
    - Postgraduate degree
    - Prefer to not say
11. What is your state of residence?

- Alabama (AL)
- Alaska (AK)
- Arizona (AZ)
- Arkansas (AR)
- California (CA)
- Colorado (CO)
- Connecticut (CT)
- Delaware (DE)
- Washington DC (DC)
- Florida (FL)
- Georgia (GA)
- Hawaii (HI)
- Idaho (ID)
- Illinois (IL)
- Indiana (IN)
- Iowa (IA)
- Kansas (KA)
- Kentucky (KY)
- Louisiana (LA)
- Maine (ME)
- Maryland (MD)
- Massachusetts (MA)
- Michigan (MI)
- Minnesota (MN)
- Mississippi (MS)
- Missouri (MO)
- Montana (MT)
- Nebraska (NE)
- Nevada (NV)
- New Hampshire (NH)
- New Jersey (NJ)
- New Mexico (NM)
- New York (NY)
- North Carolina (NC)
- North Dakota (ND)
- Ohio (OH)
- Oklahoma (OK)
- Oregon (OR)
- Pennsylvania (PA)
- Puerto Rico (PR)
- Rhode Island (RI)
- South Carolina (SC)
- South Dakota (SD)
- Tennessee (TN)
- Texas (TX)
- Utah (UT)
- Vermont (VT)
- Virginia (VA)
- Washington (WA)
- West Virginia (WV)
- Wisconsin (WI)
- Wyoming (WY)

12. To which race/ethnicity are you related? (categories according to the US Census Bureau)
    - White
    - Black or African American
    - American Indian and Alaska Native
    - Asian
    - Native Hawaiian and other Pacific Islander
    - Two or more races
    - Hispanic or Latino
    - Prefer not to say
